# Fluorescence and UV/visible spectroscopic investigation of orange and mango fruit juice quality in case of Adama Town

**DOI:** 10.1038/s41598-022-11471-7

**Published:** 2022-05-05

**Authors:** Muktar Gebishu, Boka Fikadu, Bulcha Bekele, Leta Tesfaye Jule, Nagaprasad. N, Krishnaraj Ramaswamy

**Affiliations:** 1 Department of Applied Physics, School of Applied Natural Science, Adama Science and Technology University, Adama, Ethiopia; 2 Department of Physics, College of Natural and Computational Science, Dambi Dollo University, Dambi Dolo, Ethiopia; 3 Centre for Excellence-Indigenous Knowledge, Innovative Technology Transfer and Entrepreneurship, Dambi Dollo University, Dambi Dolo, Ethiopia; 4 Department of Mechanical Engineering, ULTRA College of Engineering and Technology, Madurai, Tamil Nadu 625 104 India; 5 Department of Mechanical Engineering, College of Engineering, Dambi Dollo University, Dambi Dollo, Ethiopia

**Keywords:** Biochemistry, Biological techniques, Biotechnology, Chemical biology, Biogeochemistry, Chemistry, Energy science and technology, Engineering, Materials science, Physics

## Abstract

Extracted Mango and Orange juices were investigated by using spectroscopic techniques such as UV/Visible and Fluorescence. Three portions of samples (fresh juice) were stored at 22 °C for eight days, stored in a water bath and heated at 40 °C, 60 °C, and 80 °C for ten minutes. The highest wavelengths (455 nm) were observed from the UV/Vis results for fresh Mango juices, while 270 nm and 460 nm were observed for stored Mango juices. Furthermore, wavelengths of 320 nm were observed in heat-treated mango juice (40 °C). No absorption peaks were observed at 60 °C and 80 °C due to temperature effects. Absorption peaks of fresh fruit were observed at 330 nm and 390 nm, while 260 nm and 320 nm reflect stored orange juices absorptions peaks. From heat-treated stored (40 °C and 60 °C) samples, 320 nm and 260 nm absorption peaks were observed, respectively. Wavelength observed (454 nm, 540 nm & 700 nm) peaks represent the fresh mango juice spectra, while 460 nm and 700 nm are for stored Mango juices. The peaks observed in the region of 400–500 nm and at 700 nm represent heat-treated mango juices at 40 °C. Heat stored Mango juices (60 °C & 80 °C) have peaks at 700 nm. Peaks observed at 700 nm, 500 nm, and 455 nm reflect fresh orange juice, while 460–500 nm and 700 nm represent the emission spectra of the samples. The stored orange juice peaks at 460–500 nm and at 700 nm, but heated-stored orange juice peaks only at 700 nm. The pH values for orange and mango juices were 3.52–3.73 and 4.02–4.72, respectively.

## Introduction

Fruit quality is essential for human beings due to its excellent tests like acidness, sweetness and bitterness. These tests make feeling in the mouth of a human being due to its structures. The quality of juices has a great role in balancing factors such as acidness, sweetness, and bitterness, which are the most important factors for peoples. Particularly the corrosive and sugar substance in natural products and their proportion are exceptionally vital components for the quality of assessment by buyers^1–4^. The causticity and sugar substance are ordinarily assessed by the acidometer, bricks division which depends on refract meter and titration individually. However, anticipated esteem demonstrated as it were the general causticity and sugar substance, and don’t have sufficient truthfulness. Hence, Characterization systems were applied to get the sum and corrosive sugar. These characterization equipment are Ultraviolet, Visible spectroscopy and fluorescence^5–10^.

Natural product quality, such as colour, test, sugar substance and etc., depends on longitudinal, climate, soil, and post-harvest administration variety^10,11^. Rack lives of items are decided in duration and have an obligation as producers, administrative offices and etc. Subordinate limits on physical condition, organoleptic qualities and microbiological safety. Numerous components yonder impact duration may be categorized under natural such as water action, pH, acidity, preservatives, biochemical, microbial composition and the outward variables such as time, temperature, weight, relative mugginess, ultrasonic light, bonding fabric, and dealing with strategies^12–16^. Shelf life of most home-made items is not at all like mechanical items, predisposed to uncertainty variable and standardized. Quality of natural products such that mango and orange juice were investigated by a spectroscopic technique such as UV/Visible and Fluorescence in Japan, Denmark, and China. Still now, testing natural product juices was not investigated in Ethiopia. Therefore, the researcher wants to investigate the excellence of orange and mango fruit juices using Fluorescence and Ultraviolet–visible spectroscopy. Besides, several literatures were done on Fluorescence and UV/vis Spectroscopic Investigation of Orange and Mango Fruit Juices in different areas, especially in developed countries in order to determine the quality of juices to save the human health. But, no research was done on the quality of juices in developing countries, like Ethiopia, in areas of high temperature and several consumers of juices. Juices were prepared and settled for a few moments. This research clearly reported what happens to juices when it is fresh, stored and treated with temperatures. Thus, the main objective of this study is to investigate the quality of mango and orange fruit juice by UV/Vis and Fluorescence spectroscopy.

## Materials and methods

**Figure 1:**
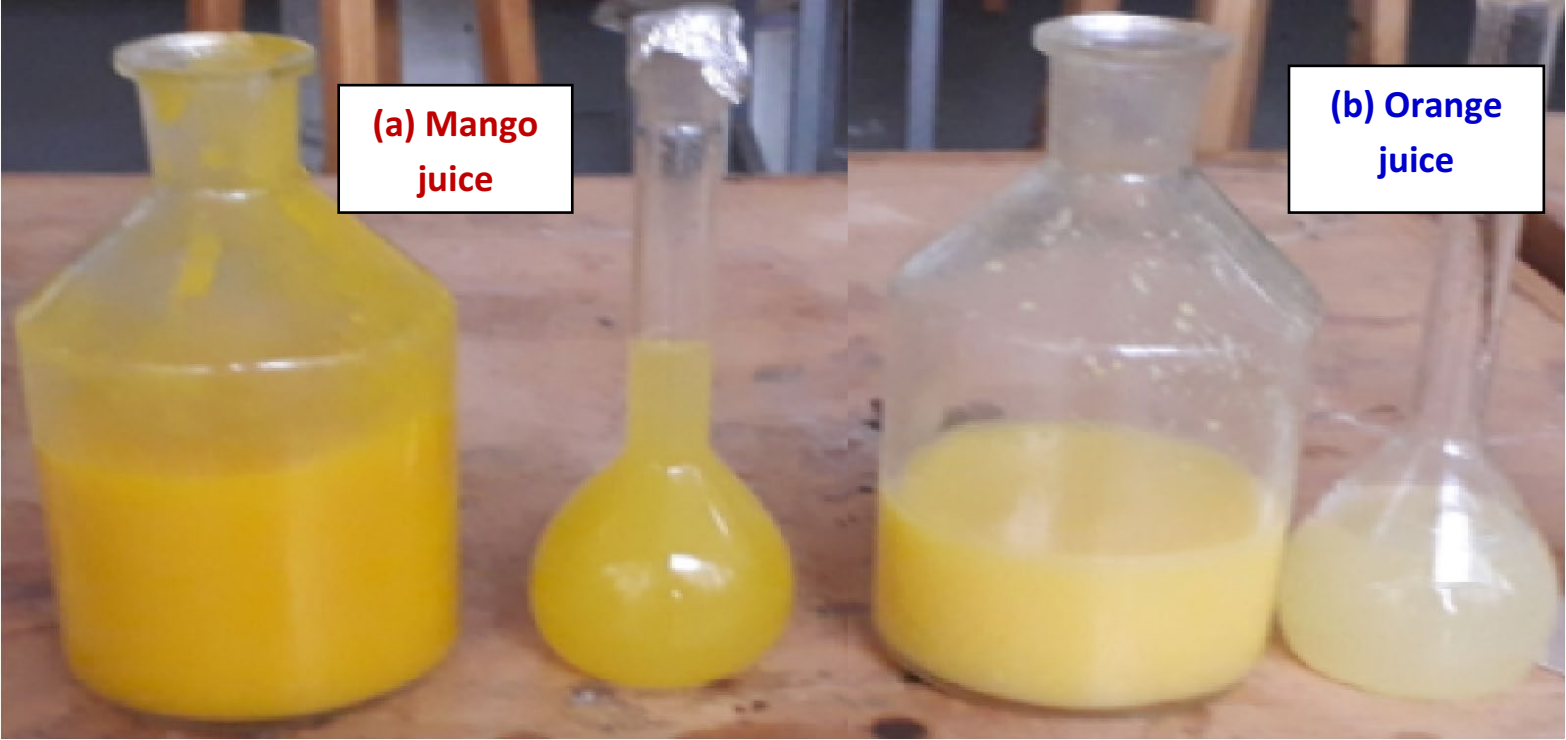
Extracted (**a**) Mango and (**b**) Orange Juice.

The explorations were carried out with the assistance of the taking after gadgets: containers, spatula, column tube, inquires, advanced electronic bar adjustments, cuvette used to take the samples for investigation, pipette used to sort, juice extractors, cone-shaped bottle, the machine connected fluorescence, and UV/vis spectrophotometer. Refrigerator and refined water were also utilized.From Literature it was reported that the pulp% for Mango Varieties (Local,Tommy,Keit,Kent,Dodo,Apple) were in the range of 65.45–78.14%^17^.since the local mango variety consists of large seed and small quality of fresh, in line to Apple Mango. Hence in this paper, Apple Mango and Orange natural product fruit juices with different colour and measures having a wide extent of colours were acquired in the city of Adama. Subsequently, the samples were protected under 6 °C temperature, and the juices were extracted with a domestic juice extractor (see Fig. 1). The natural products with comparative colour were considered as one test and extracted. Completely, natural products juice tests were arranged, and the canter was eliminated where the skin was reticent. Juices centrifuge was taken for 15 min by high rotation of 360 revolutions per minute at room temperature of 22 °C. Then, two parcels were seen by shifting the juices. One parcel was measured promptly (as fresh) and was put away at 22 °C stored for 10 days. The left parcels were warmed under the temperature of 40, 60 and 80 °C for 10 min. The juices were cooled quickly with an ice water bath and put away at 22 °C. Here, they are marked as heated stored is the reference of the measured spectra. Characterization equipment was used to test samples Fluorescence and UV/vis Spectroscopy of LAB4US UV Quartz 5 mm cuvette for spectrophotometer.The pH esteem of orange and mango juice were estimated by utilizing a pH meter where scale reading was permitted to normalize for a complete of a second. Then, a pH reading was taken. For readings, the terminals were purified with refined water and then taken to standard (pH 70 and pH 4.0) buffer solutions. Finally, data were collected from all characterization equipment and analyzed using Origin software.

## Result and discussions

**Figure 2:**
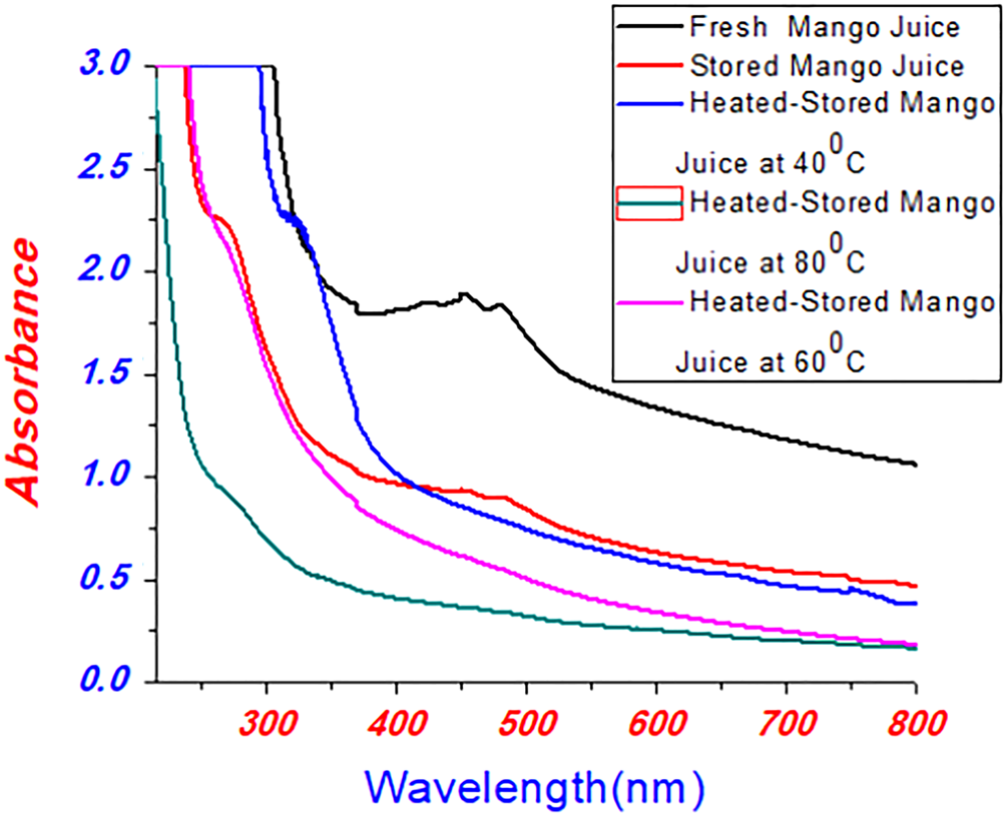
UV/Vis absorption spectra of mango juices.

This studies bargains with the investigations, discourse and the overcome of work. The quality of orange and mango juices was examined utilizing spectroscopic techniques. Specifically, absorbance spectra of Mango juice were characterized by UV/Visible spectroscopy. The mango juices assimilation spectra were measured by isolating three portions of stored mango juice and fresh mango juice. The parcel of heated, stored mango juices at 40, 60, and 80 °C were the third category. The broad peak of fresh mango juice was seen at a wavelength of 455 nm, while 270 & 460 nm was the absorption peaks for stored mango juices. The absorption peak of 320 nm represents the heat stored in mango juice. No absorption peaks were observed in heat stored mango juice of 60 °C, and 80 °C of temperatures shown in Fig. 2. This result depicts that the chemicals found in juices were destroyed at a higher annealing temperature. This reveals that annealing temperature can influence the rack life of fruit, also reported in the literature^18–20^. Additionally, coumarin was observed at absorption peas of 320 nm, whereas 270 nm represents polymethoxyavons, whiles 460 nm compares chlorophyll, also investigated in literature^14,15,21,22^.

**Figure 3:**
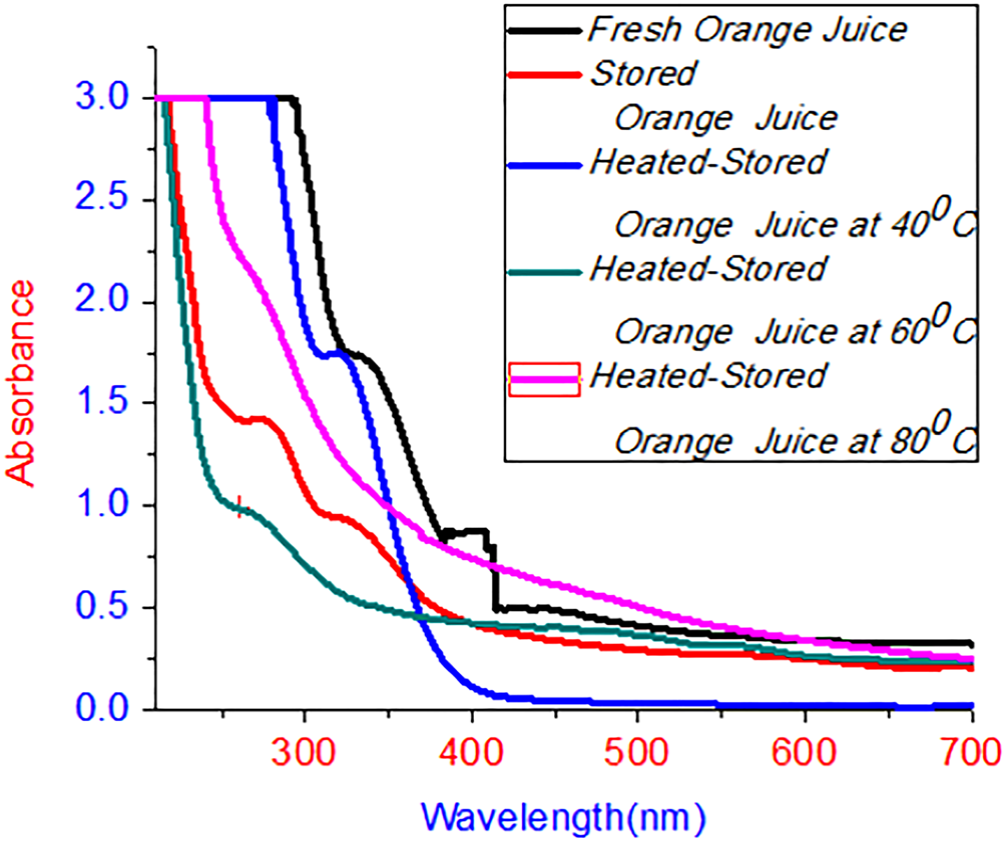
UV/vis absorption spectra of orange juice.

Figure 3 represents the UV/Vis absorption spectral of fresh, stored and heat-treated orange juices. The orange juices assimilation spectra were measured within the same methods as mango juices. The absorption peaks of fresh orange fruit were observed at 330 nm and 390 nm. Similarly, 260 nm and 326 nm represent the absorption spectra of stored orange juice. The peaks of absorption observed at a wavelength of 320 nm & 260 nm represent heat-treated or stored orange juices of 40 °C and 60 °C temperatures, respectively. There are no absorption peaks for the samples stored at a temperature of 80 °C. The absorption band found in the region of 260 to 280 nm represents vitamin C. Coumarin was found at an absorption peak of 330 nm. Coumarin could be a plant auxiliary metabolite which has capable of stopping or slowing a specific biological process. Moreover, known to be a plant controller development. The absorption peak observed at 390 nm compares vitamin A whereas 450 nm shows carotenoids^20,23–25^. Comparison stored, fresh, and heat-treated mango fruit juices with several literatures are depicted in Table 1.

**Table 1:** UV/Vis and Fluorescence Spectroscopic results of stored, fresh and heat-treated mango fruit juices compared with various literature.

Samples	UV/Vis. Spectroscopic results		References	Fluorescence spectroscopic results		References
	Absorption wavelength	Interpretations		Emission wavelength	Interpretations	
Stored Mango Juice	455 nm	Chlorophyll	^20,35^	387 nm	Carotenoids	^20,35^
Fresh Mango juice	270 nm	Polymethoxyavons	^32,33^	389 nm	Carotenoids	^20,35^
	460 nm	Chlorophyll, Coumarin	^30,35^	538 nm	Polymethoxyavons	^16,36–38^
Heat stored mango juice at 40 °C	No peak	Slightly chemicals found in juices were destroyed	^21,33,22,34,19^	386 nm	Carotenoids	^20,35^
				546 nm	Polymethoxyavons	^16,36–38^
Heat stored mango juice at 60 °C	No peak	More, chemicals found in juices were destroyed	^21,33,22,34,19^	385 nm	Carotenoids	^20,35^
				554 nm	Chlorophyll	^30,35^
Heat stored mango juice at 80 °C	No peak	Rack life of fruit is more affected	^21,33,22,34,19^	386 nm	Carotenoids	^20,35^
				555 nm	Chlorophyll	^30,35^

**Figure 4:**
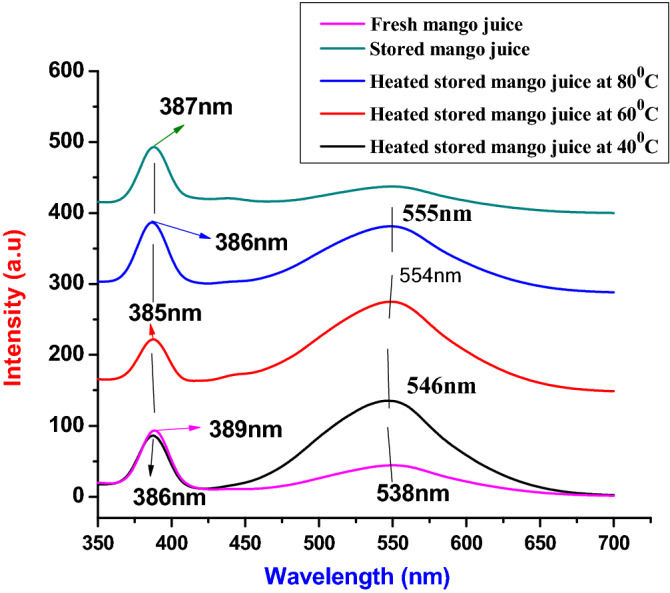
The fluorescence emission spectra of mango juices.

Figure 4 shows the emission spectra of fresh, stored and heat stored mango juices by fluorescence spectroscope. Here, the emission wavelength was adjusted in the range of 350 to 700 nm at one manometer increments, while the excitation wavelength was adjusted at 350 nm. The slit of emission was kept up under 5 nm, with the proofed speed of rotation 1200 manometer per minute, and the reaction was processed for 50 microseconds. Mango a sample was totally synthesized from stored, fresh Mango fruit juices and heat-treated stored mango fruit juices form. As depicted in Fig. 4, sharp peaks 386, 389, 385, 355 and 387 nm were the emission peaks observed in fresh mango juice, heated stored mango juice at 40 °C, heated stored mango juice at 60 °C, heated stored mango juice at 80 °C and stored mango juice respectively. Other broad peaks 538, 546, 554, and 555 nm were also seen from fresh mango juice, heated stored mango juice at 40 °C, heated stored mango juice at 60 °C, and heated stored mango juice at 80 °C, respectively. However, the emission peaks observed at 386, 389, 385, 355 and 387 nm reflect carotenoids and depict that carotenoids seen in all samples had good agreement with the result reported in the literature^16^. In addition, polymethoxyavons were seen at broad peaks of 538 nm and 546 nm, and chlorophyll was seen at emission spectra of 554 and 555 nm^20,24,26–30^.

**Figure 5:**
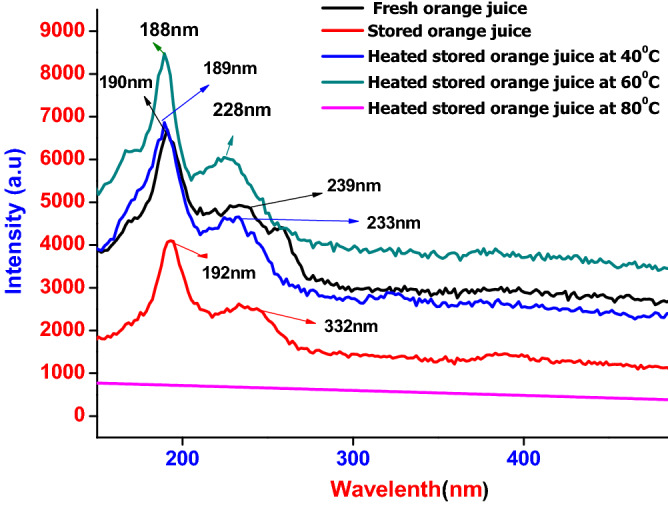
Fluorescence spectral emission of Orange juices.

The emission and excitation spectra of synthesized orange fruit juices in the condition of fresh, stored and heat store were expressed in Fig. 5, and the excitation spectra of orange fruit juices were settled at 350 nm. The emission peaks observed from fresh orange fruit juices were 190 and 239 nm, and similarly, 192 nm and 332 nm emission peaks were seen from stored orange fruit juices, which depicts overall chlorophyll. Emission peaks observed at 189 and 233 nm reflects heated stored orange fruit juices at 40 °C and express total phenol compounds. The peaks of spectral emission observed at 188 and 228 nm reflect heated stored orange juices at 60 °C and express overall carotenoids^31^. However, no emission peaks observed in the case of heated orange juices at 80 °C are due to an increment in thermal energy^19,21–23,32–34^. Comparison stored, fresh, and heat-treated orange fruit juices with several literatures are depicted in Table 2.

**Table 2:** UV/Vis and Fluorescence Spectroscopic results of stored, fresh and heat-treated orange fruit juices compared with various literature.

Samples	UV/Vis. Spectroscopic results		References	Fluorescence spectroscopic results		References
	Absorption Wavelength	Interpretations		Emission wavelength	Interpretations	
Stored orange fruit Juice	260 nm	Vitamin—C	^26,30^	192 nm	Chlorophyll	^30,35^
	450 nm	Carotenoids	^20,35^			
				332 nm		
Fresh orange fruit juice	330 nm	Coumarin	^22,34^	190 nm	Chlorophyll	^30,35^
	390 nm	Vitamin—A	^35,25^	538 nm		
Heat stored orange fruit juice at 40 °C	260 nm	Vitamin—C	^30,25^	189 nm	Phenol compounds	^30,35^
				233 nm		
Heat stored orange fruit juice at 60 °C	326 nm	Carotenoids	^31,25^	188 nm	Carotenoids	^31,35^
				289 nm		
Heat stored orange fruit juice at 80 °C	No peak	Rack life of fruit is more affected	^27,25^	No peak	Increment in thermal energy	^22,35^

**Figure 6:**
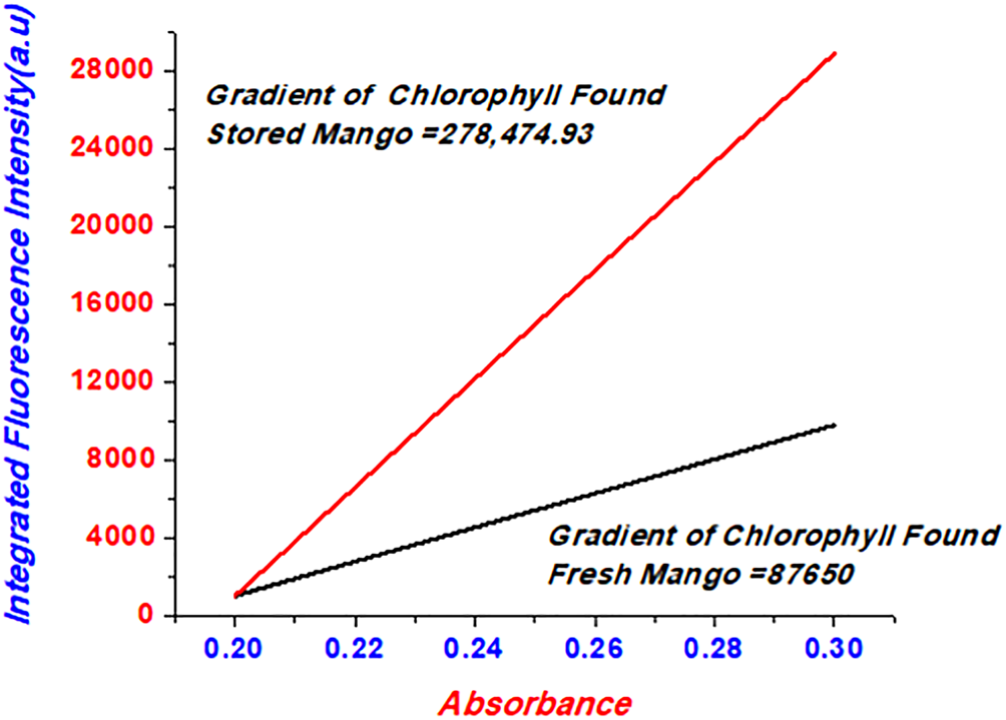
Slope of juices samples corresponding to samples of Fluorescence quantum yield chlorophyll observed in Mango Juice.

The quantum yield investigation of Mango juices was expressed in Fig. 6, and the quantum yields of chlorophyll in fresh mango juices were compared with mango juices (stored). Measurements of chlorophyll observed in Mango juices (fresh and stored) are taken as constant. Quantum abdicates, or yields of chloroplast seen in stored and fresh mango juices were determined by utilizing Eq. (1) and its esteem was 0.26.

1 $${\phi }_{F}= (1-{e}^{-\in lc})$$

The esteem of quantum surrender of chloroplast observed in mango juice (fresh) was 0.1 based on Eq. (2)

2 $${\phi }_{X}= {\phi }_{ST}\left(\frac{{Grad}_{X}}{{Grad}_{ST}}\right)\left(\frac{{{\eta }^{2}}_{X}}{{{\eta }^{2}}_{ST}}\right)$$

The index of refraction juice solvent is similar in quantum surrenders estimations is agreed with Eq. (1). The green pigments found in the chloroplast of mango juices (fresh) were considered as standard esteem of chlorophyll. They are essential, utilizing Eq. (2), the chlorophyll of quantum yield of mango juice (stored) was in the range of 0.254–0.26, which is similar to the calculated values from Eq. (1).

**Figure 7:**
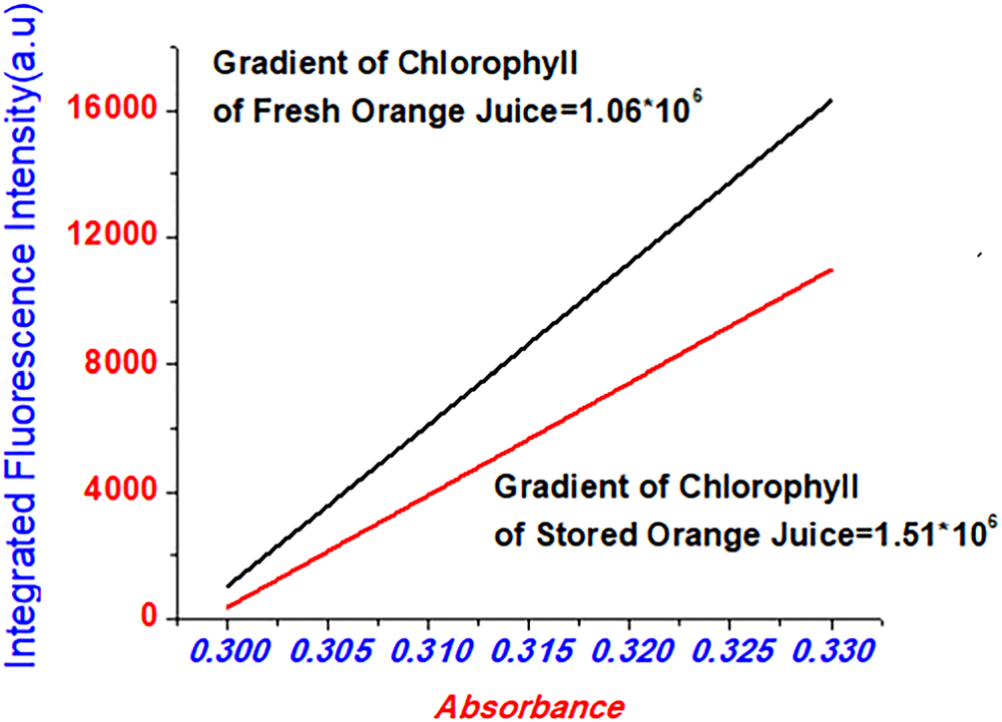
The proportionality of samples found in chlorophyll Fluorescence quantum esteem on Orange juice.

Quantum abdicates Fluorescence of Orange juice expressed in Fig. 7. The Fluorescence quantum abdicates chlorophyll found in stored and fresh orange fruit juices were the estimation. Stored Orange juices are considered standard when the chlorophyll or quantum abdicates were measured. Quantum surrender found in chlorophyll which is seen in Orange fruit (stored), was estimated utilizing Eq. (1) and its esteem was 0.27. Quantum yields observed in Orange juices (fresh) were 0.38 by utilizing Eq. (2) above in which refractive indexes of solvents quantum yield measurements are the same as refractive indexes calculated by Eq. (1). In similar steps, fresh orange juice chlorophyll is taken as standard. They are additionally utilizing conditions written in Eq. (2), quantum abdicates of chlorophyll of orange juice (stored) was 0.27.

The Orange and Mango natural products juice Fluorescence duration (lifetime) was investigated, and the sample's lifetime was measured. The result obtained from measurement was 102.8 µs which is determined from Eq. (3).

3 $${\tau }_{n}=\frac{\tau }{Q}$$

**Figure 8:**
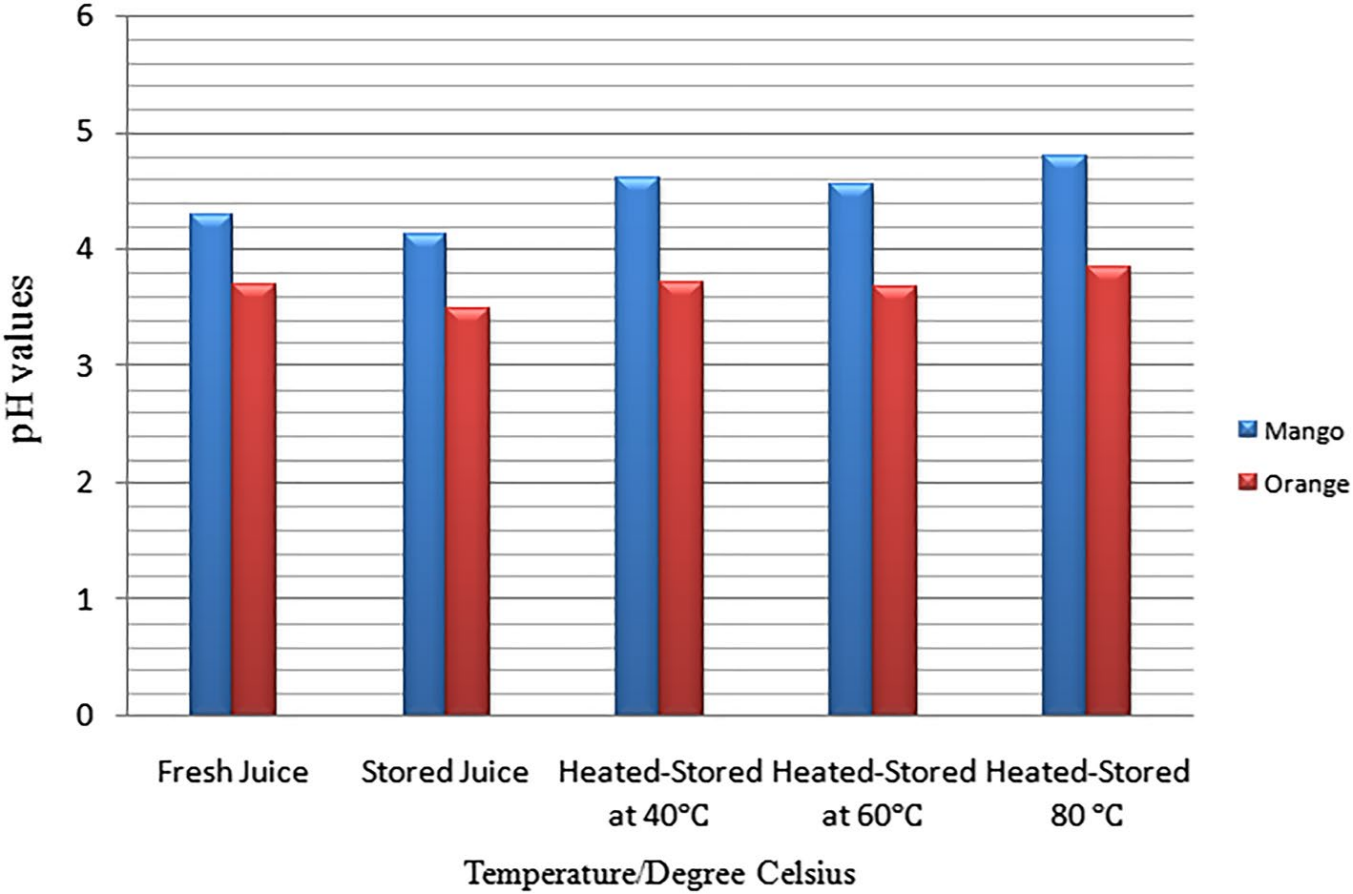
pH value of mango and orange juices.

The natural lifetime of stored mango and fresh juice is calculated utilizing Eq. (3) was 395.38 µs and 1028 µs, respectively. In addition, the time lifetimes of stored orange juice and fresh orange juice are 395.38 µs and 277.8 µs, respectively. The pH results of samples were seen in three forms, i.e. fresh mango and orange fruit, stored, and heat-treated mango and orange fruit juice at 40 °C; 60 °C; 80 °C temperatures are depicted in Fig. 8. The graph depicts that the orange fruit juice has a pH value which is less than four, which indicates strong acid when compared to mango juice^17,19–31,33–35^. Moreover, fresh, stored, the heat stored at a temperature of 40, 60 and 80 °C Orange juice has pH values of 3.7, 3.71, 3.67, and 3.85, respectively. Similarly, fresh, stored and heated stored at 40, 60, and 80 °C mango juices have the pH values of 3.49, 3.71, 3.67, 3.85, 4.65, and 4.72, respectively. This results clearly validates the comparison of pH value and lifetimes of mango and Orange fruit juices with various literature was observed in Table 3.

**Table 3:** pH value and lifetimes of mango, Orange fruit juices compared with various literature.

Fruit juice	Fresh juice	Stored juice	Heated-stored at 40 °C	Heated-stored at 60 °C	Heated-stored at 80 °C
Mango	4.3 $\pm 0.10$	4.12 $\pm 0.01$	4.6 $\pm 0.01$	4.56 $\pm 0.12$	4.80 $\pm 0.02$
Orange	3.7 $\pm 0.12$	3.49 $\pm 0.02$	3.71 $\pm 0.10$	3.67 $\pm 0.10$	3.85 $\pm 0.02$
**References**	^35,25^	^35,25^	^35,25^	^35,25^	^35,25^
	The lifetime of mango juice		The lifetime of mango juice		
	395.38		277.80		
**References**	^28,30^		^28,30^		

## Conclusions

Mango and orange fruit juice quality was investigated using VU/Vis and fluorescence spectroscopy. The UV/vis and fluorescence spectra of the prepared samples clarify the physical behaviour of fresh, stored and heat-stored mango and orange juices. The absorption ability of the sample was seen from UV/vis spectroscopy, while fluorescence gives emission properties. The broad peaks observed at 455 nm, 270 nm, 460 nm, and 320 nm represent the fresh, stored heat of mango juice at 40 °C. No absorption peaks were observed for heat-stored samples at temperatures of 60 °C and 80 °C. Here, peaks observed at 32 nm, 270 nm, and 460 nm represent coumarin, polymethoxyavons, and chlorophyll, respectively. The spectral peaks observed at 330 nm and 390 nm, and 260 and 320 nm represent fresh and stored orange juices. The peaks of the spectrum observed at heat-stored orange juice at temperatures of 40 °C and 60 °C represent 320 nm and 260 nm, respectively. No absorption peak was seen at 80 °C of temperature. Emission spectra of mango juices (fresh) were observed at 700 nm, 540 nm, and 453 nm, while 460 nm and 700 nm represent stored mango juices. The peaks observed in the region of 420 nm to 500 nm and 700 nm represent heat-treated samples (stored) at 40 °C, whereas the samples stored at 60 °C and 80 °C temperatures have spectral peaks of 700 nm. Spectral emission was observed at 700 nm, 646 nm, and 454 nm, total carotenoids, polymethoxyavons, and chlorophyll, respectively; the most intense peaks represent chlorophyll and carotenoids in these samples. Fewer carotenoids were observed at 60 °C and 80 °C, and the amount of vitamins was reduced due to heat. As observed from the results, orange juices are more influenced by temperature than mango juices. The spectroscopic investigation of fluorescence quantum yields of samples were carried out and results of 0.10 and 0.37 correspond to mango and orange juices. Additionally, the lifetime of mango and orange juices was investigated. The pH values of the juices were measured by a pH meter and resulted in regions of 4.02–4.72 and 3.52–3.73 for mango and orange juices, respectively. Using fresh juices was more important than stored, heat stored juices, and the researcher may investigate for other natural juices stored in the fridge.

## Data Availability

The data are included with in the article.

## References

[1] Haque F, Bubli SY, Khan MS (2021). UV–Vis spectroscopy for food analysis. Techniques to Measure Food Safety and Quality.

[2] Xu L, Xu Z, Liao X (2021). A review of fruit juice authenticity assessments: Targeted and untargeted analyses. Crit. Rev. Food Sci. Nutr..

[3] Giridharaprasad S, Ravi DK, Miller K, Rajoo B (2021). Optimization of incorporating κ-Carrageenan-based gels on improving cloud stability, physical stability, and viscosity of ready-to-drink mango juice. J. Food Sci..

[4] Raul PK, Santra P, Goswami D, Tyagi V, Yellappa C, Mauka V, Dwivedi SK (2021). Green synthesis of carbon dot silver nanohybrids from fruits and vegetable’s peel waste: Applications as potent mosquito larvicide. Curr. Res. Green Sustain. Chem..

[5] Sharma R, Ghoshal G (2021). Characterization and cytotoxic activity of pigment extracted from Rhodotorula mucilaginosa to assess its potential as bio-functional additive in confectionary products. J. Food Sci. Technol..

[6] Bhamore JR, Jha S, Park TJ, Kailasa SK (2019). Green synthesis of multi-color emissive carbon dots from Manilkara zapota fruits for bioimaging of bacterial and fungal cells. J. Photochem. Photobiol., B.

[7] Kiani S, Minaei S, Ghasemi-Varnamkhasti M (2016). Fusion of artificial senses as a robust approach to food quality assessment. J. Food Eng..

[8] López-Froilán R, Hernández-Ledesma B, Cámara M, Pérez-Rodríguez ML (2018). Evaluation of the antioxidant potential of mixed fruit-based beverages: A new insight on the folin-ciocalteu method. Food Anal. Methods.

[9] Raponi F, Moscetti R, Monarca D, Colantoni A, Massantini R (2017). Monitoring and optimization of the process of drying fruits and vegetables using computer vision: A review. Sustainability.

[10] Schoefs B (2002). Chlorophyll and carotenoid analysis in food products. Properties of the pigments and methods of analysis. Trends Food Sci. Technol..

[11] Masek A, Plota A, Chrzastowska J, Piotrowska M (2021). Novel hybrid polymer composites based on anthraquinone and eco-friendly dyes with potential for use in intelligent packaging materials. Int. J. Mol. Sci..

[12] Abliz A, Liu J, Mao L, Yuan F, Gao Y (2021). Effect of dynamic high pressure microfluidization treatment on physical stability, microstructure and carotenoids release of sea buckthorn juice. LWT.

[13] Adebayo WA, Ogunsina BS, Taiwo KA (2018). Sensory, textural and cooking quality of instant noodles produced from Musa spp-wheat composite flours. Arid Zone J. Eng. Technol. Environ..

[14] Wilson A, Anukiruthika T, Moses JA, Anandharamakrishnan C (2021). Preparation of fiber-enriched chicken meat constructs using 3D printing. J. Culinary Sci. Technol..

[15] Al-Degs YS (2009). Determination of three dyes in commercial soft drinks using HLA/GO and liquid chromatography. Food Chem..

[16] Roy S, Rhim JW (2021). Anthocyanin food colorant and its application in pH-responsive color change indicator films. Crit. Rev. Food Sci. Nutr..

[17] Bekele M, Sathees N, Sadik J (2020). Screening of Ethiopian mango cultivars for suitability for preparing jam and determination of pectin, sugar and acid effects on physico-chemical and sensory properties of mango jam. Sci. Afr..

[18] Abdelfatah AM, Fawzy M, El-Khouly ME, Eltaweil AS (2021). Efficient adsorptive removal of tetracycline from aqueous solution using phytosynthesized nano-zero valent iron. J. Saudi Chem. Soc..

[19] Thakker AM, Sun D (2021). Sustainable plant-based bioactive materials for functional printed textiles. J. Text. Inst..

[20] Hansawasdi C, Chaiprasart P, Meepayung P (2009). Canned mango juice processing from Mangifera indica Linn. cv Nahm-dawg-mai. Acta Horticult..

[21] Buniowska M, Carbonell-Capella JM, Znamirowska A, Zulueta A, Frígola A, Esteve MJ (2020). Steviol glycosides and bioactive compounds of a beverage with exotic fruits and stevia rebaudiana Bert. as affected by thermal treatment. Int. J. Food Prop..

[22] Mezadri T, Villaño D, Fernández-Pachón MS, García-Parrilla MC, Troncoso AM (2008). Antioxidant compounds and antioxidant activity in acerola (Malpighia emarginata DC) fruits and derivatives. J. Food Compos. Anal..

[23] Al-Yafeai A, Malarski A, Böhm V (2018). Characterization of carotenoids and vitamin E in R. rugosa and R. canina: Comparative analysis. Food Chem..

[24] Hassan ALMMI, Hazim Y (2016). Determination of Vitamin C (ascorbic acid) Contents in various fruit and vegetable by UV/Spectrophotometry and titration methods. JCPS.

[25] Khan MMR, Rahman MM, Islam MS, Begum SA (2006). A simple UV spectroscopic method for determination of vitamin C content in various fruits and vegetables at Sylhet area in Bangladesh. J. Biol. Sci..

[26] Vervoort L, Van der Plancken I, Grauwet T, Timmermans RAH, Mastwijk HC, Matser AM (2011). Comparing equivalent thermal, high pressure and pulsed electric field processes for mild pasteurization of orange juice. Part II: Impact on specifiec chemical and biochemical quality parameters. Innov. Food Sci. Emerg. Technol..

[27] Wibowo S, Vervoort L, Tomic J, Santiago JS, Lemmens L, Panozzo A, Grauwet T, Hendrickx M, Loey, A.V.  (2015). Colour and carotenoid changes of pasteurized orange juice during storage. Food Chem..

[28] Falade KO, Babalola SO, Akinyemi SOS, Ogunlade AA (2004). Degradation of quality attributes of sweetened Julie and Ogbomoso mango juices during storage. Eur. Food Res. Technol..

[29] Mahdavi R, Nikniaz Z, Rafraf M, Jouyban A (2010). Determination and comparison of total-polyphenol and vitamin C contents of natural fresh and commercial fruit juice. Pak. J. Nutr..

[30] Fratianni A, Cinguanta L, Panfill G (2015). Degradation of carotenoids in orange juice during microwave heating. LWT-Food Sci. Technol..

[31] Alaka OO, Aina JO, Falade KO (2003). Effect of storage conditions on the chemical attributes of Ogbomoso mango juice. Eur. Food Res. Technol..

[32] Neves LC, Tosin JM, Benedette RM, Cisneros-Zevallos L (2015). Post-harvest nutraceutical behaviour during ripening and senescence of 8 highly perishable fruit species from the Northern Brazilian Amazon region. Food Chem..

[33] Eskandari T, Niazi A, Fatemi MH, Chaichi MJ (2021). Determination of fenthion in environmental water samples by dispersive liquid–liquid microextraction coupled with spectrofluorimetric and chemometrics methods. Anal. Methods Environ. Chem. J..

[34] Way ML, Jones JE, Swarts ND, Dambergs RG (2019). Phenolic content of apple juice for cider making as influenced by common pre-fermentation processes using two analytical methods. Beverages.

[35] Santos DA, Lima KP, Marco PH, Valderrama P (2016). Vitamin C determination by ultraviolet spectroscopy and multiproduct calibration. J. Braz. Chem. Soc..

[36] Nguenha R, Damyeh MS, Phan AD, Hong HT, Chaliha M, O’Hare TJ, Sultanbawa Y (2021). Effect of photosensitization mediated by curcumin on carotenoid and aflatoxin content in different maize varieties. Appl. Sci..

[37] Fan K, Zhang M (2019). Recent developments in the food quality detected by non-invasive nuclear magnetic resonance technology. Crit. Rev. Food Sci. Nutr..

[38] Hempel J, Schädle CN, Sprenger J, Heller A, Carle R, Schweiggert RM (2017). Ultrastructural deposition forms and bioaccessibility of carotenoids and carotenoid esters from goji berries (Lycium barbarum L.). Food Chem..

